# Absence of Anti-Glomerular Basement Membrane Antibodies in 200 Patients With Systemic Lupus Erythematosus With or Without Lupus Nephritis: Results of the GOODLUPUS Study

**DOI:** 10.3389/fimmu.2020.597863

**Published:** 2020-12-14

**Authors:** Nellie Bourse Chalvon, Pauline Orquevaux, Delphine Giusti, Gregory Gatouillat, Thierry Tabary, Marcelle Tonye Libyh, Jan Chrusciel, Moustapha Drame, Grace Stockton-Bliard, Zahir Amoura, Laurent Arnaud, Hanns-Martin Lorenz, Gilles Blaison, Bernard Bonnotte, Nadine Magy-Bertrand, Sabine Revuz, Reinhard Edmund Voll, Oliver Hinschberger, Andreas Schwarting, Bach Nga Pham, Thierry Martin, Jean-Loup Pennaforte, Amelie Servettaz

**Affiliations:** ^1^ Département de médecine interne, Centre Hospitalier Universitaire de Reims, Reims, France; ^2^ Département d'immunologie biologique (laboratoire d'immunologie), Centre Hospitalier Universitaire de Reims, Reims, France; ^3^ Département d'information médicale et d'évaluation des performances, santé publique, Centre Hospitalier de Troyes, Troyes, France; ^4^ Département de Délégation à la Recherche Clinique et à l'Innovation, University Hospital of Martinique, Fort-de-France, Martinique; ^5^ Commission de la recherche, Université de Reims Champagne-Ardenne, Reims, France; ^6^ Service de Médecine interne, Assistance Publique Hôpitaux de Paris (APHP), Paris, France; ^7^ Service de Rhumatologie, Hôpitaux Universitaires de Strasbourg, Strasbourg, France; ^8^ Division of Rheumatology, Clinic for Hematology, Oncology and Rheumatology, Department of Internal Medicine V, University Hospital Heidelberg, Heidelberg, Germany; ^9^ Département de médecine interne, Hôpital Pasteur, Colmar, France; ^10^ Département de Médecine Interne et d’immunologie Clinique, Centre Hospitalier Regional Universitaire De Dijon, Dijon, France; ^11^ Département de médecine interne, Centre Hospitalier Universitaire de Besançon, Besancon, France; ^12^ Département de médecine interne, Hôpital Belle-Isle, Metz, France; ^13^ Department of Rheumatology and Clinical Immunology, Medical Center, University of Freiburg, Freiburg, Germany; ^14^ Département de médecine interne, Groupe Hospitalier de la Région de Mulhouse et Sud Alsace (GHRMSA), Mulhouse, France; ^15^ Universitäres Centrum für Autoimmunität Mainz, Johannes Gutenberg-Universität Mainz, Mainz, Germany; ^16^ Immunologie Clinique et Médecine Interne, Hôpitaux Universitaires de Strasbourg, Strasbourg, France

**Keywords:** anti-glomerular basement membrane antibodies, lupus nephritis, Goodpasture disease, anti-GBM antibodies, anti-GBM glomerulonephritis, systemic lupus erythematosus

## Abstract

**Introduction:**

Anti-glomerular basement membrane (GBM) antibodies are pathogenic antibodies first detected in renal-limited anti-GBM disease and in Goodpasture disease, the latter characterized by rapidly progressive crescentic glomerulonephritis combined with intra-alveolar hemorrhage. Studies have suggested that anti-GBM antibody positivity may be of interest in lupus nephritis (LN). Moreover, severe anti-GBM vasculitis cases in patients with systemic lupus erythematosus (SLE) have been described in the literature, but few studies have assessed the incidence of anti-GBM antibodies in SLE patients.

**Objective:**

The main study objective was to determine if positive anti-GBM antibodies were present in the serum of SLE patients with or without proliferative renal damage and compared to a healthy control group.

**Methodology:**

This retrospective study was performed on SLE patients’ sera from a Franco-German European biobank, developed between 2011 and 2014, from 17 hospital centers in the Haut-Rhin region. Patients were selected according to their renal involvement, and matched by age and gender. The serum from healthy voluntary blood donors was also tested. Anti-GBM were screened by fluorescence enzyme immunoassay (FEIA), and then by indirect immunofluorescence (IIF) in case of low reactivity detection (titer >6 U/ml).

**Results:**

The cohort was composed of 100 SLE patients with proliferative LN (27% with class III, 67% with class IV, and 6% with class V), compared to 100 SLE patients without LN and 100 controls. Patients were mostly Caucasian and met the ACR 1997 criteria and/or the SLICC 2012 criteria. Among the 300 tested sera, no significant levels of anti-GBM antibodies were detected (>10 U/ml) by the automated technique, three sera were found “ambivalent” (>7 U/ml): one in the SLE with LN group and two in the SLE without LN group. Subsequent IIF assays did not detect anti-GBM antibodies.

**Conclusion:**

Anti-GBM antibodies were not detected in the serum of Caucasian patients with SLE, even in case of renal involvement, a situation favoring the antigenic exposure of glomerular basement membranes. Our results reaffirm the central role of anti-GBM antibodies as a specific diagnostic biomarker for Goodpasture vasculitis and therefore confirm that anti-GBM antibody must not be carried out in patients with SLE (with or without LN) in the absence of disease-suggestive symptoms.

## Introduction

Lupus nephritis (LN) is a classic complication, and occurs in approximately 41% of European systemic lupus erythematosus (SLE) patients, and respectively 43 to 80% of African and Asian SLE patients (1), usually during the first years of the disease. Renal biopsy determines histological classification, prognosis evaluation and thus helps guide treatment (2–4). Regular screening for renal damage is thus recommended, using various biomarkers, such as microhematuria, proteinuria, complement level, anti-DNA antibody positivity, or serum creatinine associated with glomerular filtration rate. Quantification of proteinuria is described as the most sensitive biomarker for screening SLE renal involvement, with massive proteinuria being reported in 75% of class IV glomerulonephritis (5). New biomarkers have been proposed and are currently under study (genetic, epigenetic, auto-immune, and/or proteinaceous markers). However they are not yet accessible in current practice and their interpretation is a source of controversy (6–8). Some studies have suggested that anti-glomerular basement membrane antibodies (anti-GBM) may be of clinical relevance in SLE and the detection of LN (9).

Anti-glomerular basement membrane (GBM) antibodies are pathogenic antibodies, first detected in renal-limited anti-GBM disease and in Goodpasture disease, a rare vasculitis affecting approximately 0.2 to 1 case per million population (10, 11) and causing rapidly progressive renal failure due to crescentic glomerulonephritis (CGN), with linear deposits of IgG along the GBM, and intra-alveolar hemorrhages (IAH) (12, 13). The pathogenic nature of anti-GBM antibodies has been confirmed and involves a complement-dependent cytotoxicity mechanism. These antibodies target the noncollagenous-1 (NC1) domain of the α3 chain of collagen IV of GBM (14). Their synthesis is known to be stimulated in pathologic renal situations which cause GBM antigenic exposure (15, 16).

Cases of patients presenting both SLE and anti-GBM vasculitis have been reported in medical literature, including one case in our internal medicine department (17–20). Most of these patients were young and hospitalized in serious condition with rapidly progressing renal failure [with either only anti-GBM glomerulonephritis or mixed damage associating (type IV) LN lesions and anti-GBM vasculitis lesions in their kidney histology] and/or intra-alveolar hemorrhage. In 2006, a Chinese retrospective cohort reported a relatively high rate of positive anti-GBM antibodies in SLE patients [n = 14/157 (8,9%)] and amongst patients with LN, histological damage was more serious in those with positive anti-GBM antibodies (9). Furthermore, some experimental studies have shown that some immunological and genetic mechanisms were common to both pathologies (21–24). These elements raise the possibility of a GBM-SLE overlap syndrome, similar to antineutrophil cytoplasmic antibody (ANCA)-associated vasculitis (SLE/AAV) overlap syndrome (25–27). However, the results of other studies published on anti-GBM antibody detection are contradictory and did not show higher seropositivity in SLE patients (28–30). Indeed, these anti-GBM antibodies are known to be highly specific for vasculitis within the overall Caucasian population (10, 11). Other than in renal-limited anti-GBM and Goodpasture diseases, anti-GBM antibodies have been frequently observed in anti-neutrophil cytoplasmic antibody (ANCA)-associated vasculitis. Importantly, these double-positive ANCA and anti-GBM vasculitis appear to combine the demography and extra-renal and pulmonary involvement seen in ANCA-associated vasculitis with the histological type and severe renal prognosis of anti-GBM vasculitis (31–36).

To search for possible links between anti-GBM antibodies, SLE, and LN, our study therefore aimed to assess the incidence of anti-GBM auto-antibodies in SLE (associated or not with LN). In the event of a positive result, the relation between anti-GBM positivity and the histological severity of LN renal damage would be analyzed.

## Patients and Methods

### Presentation of the LBBR Lupus Biobank and Experimental Scheme of the Study

This retrospective study called « *Goodlupus project* » was carried out using the Lupus Biobank of the upper Rhine (LBBR), a large German-French cohort of SLE patients (37). Seventeen different university and general hospitals in France and Germany helped to build this biobank between 2011 and 2014 including Strasbourg University Hospital, Colmar University Hospital; Mulhouse General Hospital, Nancy General Hospital, Metz private hospitals, Belfort General Hospital, Dijon University Hospital, Besançon University Hospital, Reims University Hospital, Paris-Salpetrière University Hospital, Lyon University Hospital, Johannesburg Gutenberg de Mainz University Hospital, the Central Karlsruhe Clinic, Heidelberg University Hospital, Baden Baden Hospital and Freiburg University hospital. LBBR was created to develop various research projects devoted to SLE, with a focus either on genetics, new prognostic biomarkers, or new therapeutic measures for this illness.

On the day of inclusion in the LBBR Biobank, a blood sample was taken, and clinical data were gathered using a standard form completed by the investigators. Sample and forms were subsequently linked anonymously to build an extensive data base. To be included in the LBBR cohort, patients had to meet the following criteria: aged >16 years, have seen a doctor in one of the 17 participating centers, fulfil the ACR 1997 (38) and/or SLICC 2012 (39) criteria for SLE, and have provided written informed consent. Patients were included irrespective of the activity level of their SLE at consultation, even if inactive. Samples were sent within 24 h to Strasbourg or Freiburg, aliquoted and duplicated, then frozen and stored in both Strasbourg and Freiburg (to secure the biobank). Clinical data were anonymized (using an identification number linking the sample to the patient’s data) and computerized in a secure databank. A scientific committee had been established previously to oversee the use of the LBBR biobank.

For the patients included in the *Goodlupus project*, the criteria were the following: inclusion in the LBBR cohort (and thereby have a corresponding sample and data form). For the patients of the LN group: at least one renal biopsy showing class III, IV, or V LN damage of the ISN classification (2). The non-inclusion criteria included: lack of data regarding renal damage or patients diagnosed with class I, II, or VI LN of the ISN classification (2).

The control group was made up of sera from healthy volunteer blood donors paired for gender with the patients. Controls had to fulfill the basic requirements for blood donation and meet the following criteria: aged >18 years, no renal damage or chronic auto-immune disease, not taking immunosuppressive medication, and a signed written consent form.

### Legal Provisions and Ethical Considerations

In regard to the use of patient sera and computerized medical data from the LBBR biobank, the project had received prior to the study onset authorization from both the French Ethical Research Committee—”*Comité de Protection des Personnes*” (CPP Est 2.02.2011), and the French National Commission for Information Technology and Civil Liberties (CNIL; n° CERFA 13809*02). The investigators did not have any access to patient personal information (date of birth, initials etc.).

For the healthy volunteer blood donor sera, the study was approved by a regional ethics committee (CPP, Sud-Est IV**)** on March 16th, 2018 and an agreement was signed between the “*Établissement Français du Sang*” (EFS, French National Blood Services), Reims, and the Reims University Hospital, specifically for this study. Every healthy volunteer participating in the study was required to provide written consent and have health coverage.

### Assessment and Evaluation Criteria

Data gathered included: the proportion of seropositive anti-GBM patients in each group; age of patients and controls at blood sampling (i.e. at inclusion in the LBBR or when blood was sampled). For patients, the clinical and biological features of their SLE in the 28 days preceding the serum sampling and their disease activity [assessed by the 1992 SLEDAI disease activity index (40) and the *“medical assessment scoring”*] were assessed using the LBBR data. Age at SLE diagnosis, ethnicity, and the main clinical and biological features of the disease since diagnosis were also gathered from the LBBR Biobank. For cytopenia, the thresholds were the following: neutropenia if the neutrophil ratio was <1,800/mm3, lymphopenia if the lymphocyte count was <1,500/mm3, thrombocytopenia if the platelet count was under 100,000/mm3. Hemolytic anemia was considered present with a positive anti-globulin test. The presence of antinuclear antibodies (ANAs) (by immunofluorescence on HEP2 cells) was confirmed if fluorescence was visible at dilution under 1/160th. These data were taken from the LBBR database described above.

### Biological Analyses and Dosage Method for Anti-GBM antibodies

Anti-GBM antibodies were sought in the LBBR biobank serum samples using at least two different techniques. The frozen sera at the Strasbourg lab were brought on dry ice to the immunology lab of Reims University Hospital where the analysis was performed after onsite defrosting. A first screening was performed using a fluoro-immunoenzymatic (FEIA) type automated technique that identified the serum noncollagenous-1 (NC1) domain of the α3 chain of collagen IV antibodies (ELiA GBM^®^ test on the immuno-CAP250^®^, sold by Thermoscientific^®^ of the Phadia^®^ labs). In accordance with the supplier’s recommendations, the result was considered positive when the concentration detected was above 10 U/ml. Equivocal results (detection between 7 and 10 U/ml), or positive (>10 U/ml) were followed by a second indirect immunofluorescence (IIF) assay. This second method was performed on 6M urea-treated primate kidney sections, using a FITC-labeled anti-human IgG class conjugate with an initial dilution of 1:10 [NOVA Lite^®^ GBM (primate kidney) and GBM antigens sold by the Werfen^®^ labs]. This second assay detected autoantibodies targeting glomerular basement membrane antigens including the NC1 region of the alpha-3 chain of the network-structured type IV collagen. A third assay by immunodot (GA Generic Assays GmbH^®^) was performed when the first two were discordant. This latter immunoassay detected human antibodies directed against the NC1 domain of the α3 chain of collagen IV.

**Figure 1:**
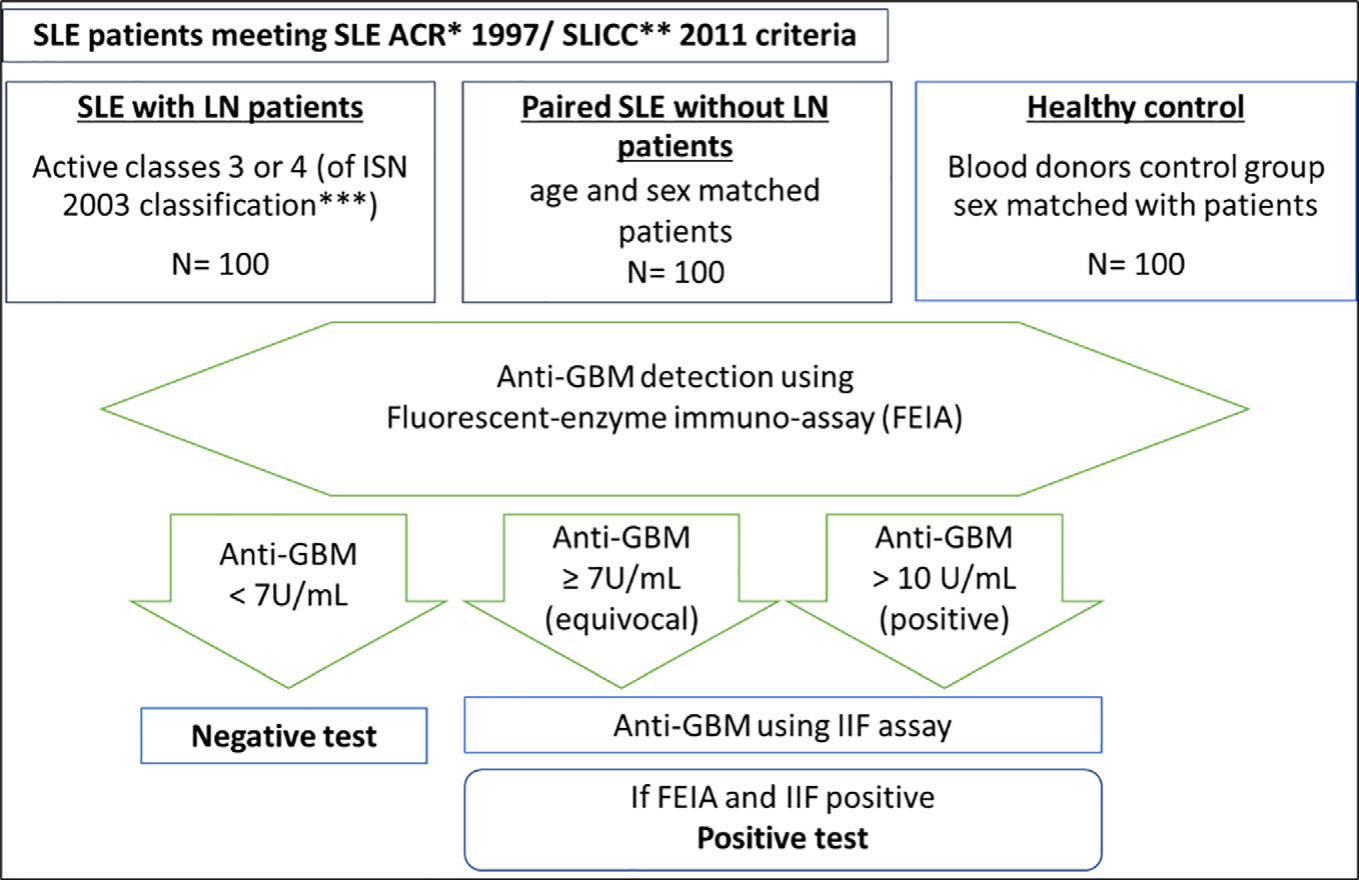
Approach and techniques used to test for serum anti-GBM antibodies in the immunology laboratory of Reims University Hospital. *ACR criteria: SLE classification criteria of the American College of Rheumatology (38). **SLICC criteria: (39). *** 2003 classification of lupus nephritis if the International Society of Nephrology (2).

A serum was considered positive only with the appearance of anti-GBM antibodies for two dosage techniques. The analyses were performed on the samples within 72 h after defrosting, and the sera were kept in the fridge (at +4°C) in the meantime (**Figure 1**).

### Statistical Analyses

#### Sample Size Determination

The calculation of the number of subjects needed per group was based on Li et al.’s study where 8.9% of patients with LN had positive anti-GBM (9) using the 1978 Casagrande-Pike-Smith approximate sample-size formula (41). It was calculated that 65 patients per group were needed to demonstrate an association between LN and anti-GBM antibodies, with an 80% power, and an alpha risk of 0.05. However, to increase the statistical power of the study, the *LBBR scientific committee* decided to include 100 subjects per group.

#### Analyzing the Main Evaluation Criterion

The qualitative variables were described by the matching headcounts and frequencies, and the quantitative variables by their average and standard deviations. We had initially planned to compare the ratio of patients presenting positive anti-GBM antibodies in each group using a chi-2 test. To identify correlations between the sociodemographic, clinical, biological, and immunological characteristics and the presence of serum anti-GBM antibodies, univariate analyses were proposed with a threshold of p < 0.020.

## Results

### General Characteristics of Patient and Control Group Populations

Three hundred subjects (100 SLE with LN, 100 SLE without LN, paired according to age and gender, and 100 age-matched voluntary blood donors) were included. The mean age at the time of the blood sample was 40 ( ± 13) years old for the 200 SLE patients, and 35 ( ± 14) years old for the healthy control group (p = 1). In the three groups, the sex ratio was 8 women for 2 men. Patient age at SLE diagnosis did not significantly differ according to the presence or not of renal damage during follow-up. There were statistically more Caucasian patients in the SLE group without LN than in the SLE group with LN [88% (n = 78/88) *vs.* 72% (n = 42/58); p = 0.02], and more patients of North-African origin in the LN group than in the SLE group without LN [13% (n = 8/58) *vs.* 1% (n = 1/88); p = 0.02]. Other ethnic origins were poorly represented, mainly Asian ethnicity [3% (n = 2/58) in the SLE group with LN and 3% (n = 3/88) in the SLE group without LN].

### Clinical, Biological, and Histological SLE Characteristics Since Disease Diagnosis

Among the SLE with LN patient group, histological damage was mainly class III [27% (n = 27/100)] and IV [67% (n = 67/100)] glomerulonephritis, of which 25% (n = 25/100) was of class IV-S (diffuse segmental damage) and 42% (n = 42/100) of class IV-G (diffuse global damage) according to the ISN classification (**Table 1**).

**Table 1:** Renal, clinical, hematological, and immune characteristics presented by patients since SLE diagnosis.

	SLE with LN *No (%)*	SLE without LN *No (%)*	*p*
**Lupus nephritis** (classification of renal histologies—ISN/RPS) **– n (%)**	100/100 (100)	0/100 (0)	<0.001
Class III – focal glomerulonephritis	27/100 (27)	NA	NA
Class IV – a) 4-S Diffuse segmental glomerulonephritis	25/100 (25)	NA	NA
Class IV – b) 4-G Diffuse glomerulonephritis	42/100 (42)	NA	NA
Class 5 – Extra-membranous glomerulonephritis	6/100 (6)	NA	NA
**Clinical SLE involvement – n (%)**			
Joint damage	61/100 (61)	72/100 (72)	0,1
Skin damage Discoid lupus	20/100 (20)	16/98 (16)	0,50
Malar rash	54/100 (54)	52/100 (52)	0,78
Oral ulcerations	27/99 (27)	15/100 (15)	0,34
Photosensitivity	49/100 (49)	60/100 (60)	0,12
Neurological damage	10/100 (10)	9/99 (9)	0,83
Serous inflammation (pericarditis/pleurisy)	36/99 (36)	15/100 (15)	<0,001
**SLE hematological disease – n (%)**			
Positive anti-globulin direct test	30/77 (38)	19/50 (38)	0,91
Lymphopenia (<1,500/mm3)	48/100 (48)	60/100 (60)	0,89
Neutropenia (<1,800/mm3)	20/99 (20)	24/100 (24)	0,52
Thrombocytopenia (<100,000/mm3)	17/98 (17)	24/99 (24)	0,23
**SLE autoimmune serological markers – n (%)**			
Positive antinuclear antibodies using IFI (>1/160th)	60/64 (93)	87/94 (92)	0.71
Positive anti-DNA at least once	94/100 (94)	59/100 (59)	<0.001
Positive anti-nucleosomes	16/39 (41)	21/70 (41)	0,24
Positive anti-SSA	21/59 (35)	38/87 (43)	0,38
Positive anti-Sm	16/97 (16)	8/84 (9,5)	0,26
Low complement level at least once	43/92 (46)	46/78 (63)	0,19

SLE, systemic lupus erythematosus; LN, lupus nephritis; ISN/RPS, international classification for LN (2); NA, non-appropriate; IFI, indirect immunofluorescence; LN, Lupus nephritis; SLE, systemic erythematosus lupus; SD, standard deviation.

Extra-renal clinical signs reported since SLE diagnosis are presented in **Table 1**. In the LN group, 61% (n = 61/100) had presented joint damage, 20% (n = 20/100) had presented discoid lupus, 10% (n = 10/100) had or had presented in the past central and/or peripheral neurological damage. Only the serositis incidence throughout the course of the SLE was different between both groups [36% (n = 36/99) in the group with LN and 15% (n = 15/100) in the group without LN respectively (p < 0.001)]. Regarding laboratory features, cytopenias were comparable in both groups.

Regarding the immunological profile (**Table 1**), patients from the SLE with LN group were more likely to have had a history of positive anti-DNA antibodies than patients from the SLE without LN group [94% (n = 94/100) of positive anti-DNA at least once in the SLE group with LN *versus* 59% (n = 59/100) in the group without LN; p < 0.001]. The search for ANAs came back positive using immunofluorescence (titer >1/160th) in more than 90% of both groups. The specificities of antinuclear antibodies did not differ between the two groups of patients for SSA, SSB, Sm, and nucleosomes. No significative difference was observed between the two groups’ antiphospholipid antibody frequency.

### SLE Clinical Activity and Treatment on the Day of Blood Sampling

The SLE clinical activity on the day of blood sampling, assessed using the international SLEDAI score, was on average higher in the SLE group with LN [SLEDAI 6,28 ( ± 4.6)] as compared to patients without LN [SLEDAI 3,19 ( ± 2.3)]; p<0.0001 [IC 95% (1.74; 4.43)] (**Table 2**). The numerical activity score (rating from 0 to 3 depending on the clinical evaluation by the specialist) was 1.025 on average in the SLE group with LN and 0.73 in the SLE group without LN; p = 0.06.

**Table 2:** SLE activity and treatment in the 28 days before blood sampling.

	SLE with LN *No (%)*	SLE without LN *No (%)*	*p*
**Global SLE disease activity—average (SD)**			
Mean activity according to SLEDAI score at sampling	6,28 ( ± 4.6)	3,19 ( ± 2.3)	<0,001
Physician’s global assessment of disease activity^*^	1,02	0,73	0.06
**Medical treatment the day of blood sampling—n (%)**			
Azathioprine	8/59 (13)	12/96 (12)	0.78
Belimumab	1/58 (1)	1/96 (1)	NS
Cyclophosphamide	12/61 (19)	2/94 (2)	0.02
Hydroxychloroquine	41/64 (64)	72/97 (74)	0.04
Mycophenolate MOFETIL	39/61 (63.9)	12/95 (12.6)	<0,001
Rituximab	1/59 (1.7)	3/95 (3.0)	NS
Azathioprine	8/59 (13)	12/96 (12)	0.78
Corticosteroids			
Under 10 mg per day	32/63 (50.8)	36/95 (37.9)	0.48
Above 10 mg per day	19/63 (30.1)	15/95 (15.8)	0.03
No corticosteroids	12/63 (19.0)	44/95 (46.3)	<0.001

LN, lupus nephritis; SLE, systemic lupus erythematosus; SD, standard deviation.

SLEDAI, Systemic Lupus Erythematosus Disease Activity Index (40), no missing data.

*Global assessment score of disease activity according to clinician (activity score, graded between 0 and 3 depending on the clinician’s assessment of the SLE activity on the day of the blood sampling, 0 being inactive and 3 being highly active). For the assessment score: 40 answers were available in the group with LN, and 49 in the group without LN in the LBBR biobank database. NS, non-significant.

Regarding medical treatments (**Table 2**), hydroxychloroquine was prescribed to 64% (n = 41/64) of patients in the SLE group with LN and 74% (n = 72/97) of patients in the SLE group without LN at database inclusion. As expected, patients in the SLE group with LN received statistically more immunosuppressants on the day of blood sampling than those of the SLE without LN group.

### Anti-GBM Antibody Detection

The mean concentration of anti-GBM antibodies was measured using the FIEA technique (ELiA GBM^®^ tests on *immuno-CAP250^®^* automaton marketed by the *Phadia^®^ labs*) in the three groups. No serum from any subject contained anti-GBM antibodies above the positivity threshold validated for the technique (concentration >10 U/ml). One serum from the SLE group with LN, and two sera from the SLE group without LN did however contain a concentration of anti-GBM antibodies above 7 U/ml. Further assays for anti-GBM antibodies using IIF and immunodot were therefore conducted on these three sera, which came back strictly negative.

## Discussion/Conclusion

In this study of 200 SLE patients, no positive anti-GBM antibodies were identified in the serum. To our knowledge, this is largest anti-GBM antibody study ever conducted on SLE and histologically demonstrated LN.

This study was performed because several cases of anti-GBM antibody vasculitis in SLE patients have been reported in the literature (four cases are presented in **Table 3**) (17–20), and because some studies suggest a possible interest in the dosage of anti-GBM antibodies in LN. In a Chinese study, Li et al. found that 8.9% (n = 14/157) of SLE patients had positive anti-GBM antibodies identified using ELISA (EUROIMMUN^®^ kit), and 8.3% (n = 13/157) were also positive using IIF (9). The frequency of anti-GBM antibodies therefore seemed to be high in SLE patients of Asian descent. Furthermore, the 14 SLE patients with positive anti-GBM antibodies also presented LN. However, most of these “LN with anti-GBM antibodies” patients in this study matched the diagnostic criteria for anti-GBM vasculitis (n = 5/14), and most of them presented alveolar hemorrhages [64.3% (n = 9/14) *versus* 1.4% (n = 1/74) of the “LN without anti-GBM antibodies” group; p<0.001] (13). Moreover, patients with “LN with anti-GBM antibodies” had a worse histological damage prognosis than the LN patients without anti-GBM antibodies of this Chinese cohort. Interestingly, in the cases reported in the literature (**Table 3**), a poor renal prognosis was observed in the two SLE patients with anti-GBM antibodies and linear IgG deposits (with concurrent class IV LN in one patient), whereas renal function recovery was reported in the other SLE patient with anti-GBM antibodies but without any IgG deposits on kidney biopsy. This may suggest that two distinct renal profiles may occur when SLE and anti-GBM antibodies coexist characterized by distinct histological hallmarks and prognosis.

**Table 3:** Review of SLE patient cases and anti-GBM antibodies reported in the literature.

First author, year of publication, magazine/Journal	Patient’s gender*(origin of the case)*	Age (years)	Technique used for searching anti-GBM in the serum	ELISA anti-GBM titer (U/ml)	Severe renal failure *(initial)*	IAH*(initial)*	Time lapse between the severe episode and the renal biopsy	Histological aspect of LN*(MO and IF)*	Linear IgG deposits along the GBM	ACR criteria of SLE		Treatment/*Evolution*
										Clinical	Biological	
*T. Yamada et al*., 2018, *J. Nippon Med Sch* (17)	Female *(Japan)*	42	IIF and ELISA *(ND)*	11,9	Yes	Yes	19 days after hospital admission	Yes	No	Skin damage, arthralgias, and LN	Anti-DNA positivity (14UI) and ANA, hypocomplementemia	Methylprednisolone (three boluses) and plasma exchanges/*normalization of the renal function and IAH decrease*
*N. Chalvon et al*., 2015, *La revue de médecine interne* (18)	Female *(France)*	31	IIF and ELISA (Chemi-luminescence using *Bioflash^®^)*	1,100	Yes	Yes	2 days (after treatment onset)	No	Yes	Skin damage, arthralgias, and AITP	Anti-DNA positivity (21UI) and ANA, hypocomplementemia	Methylprednisolone (three boluses), plasma exchanges followed by cyclophosphamide/ *chronic terminal renal failure (hemodialysis), IAH decrease*
*M. Yalda et al*., 2011, *J. Kidney Dis. Transpl* (19)	Male *(USA)*	32	IIF and ELISA *(technique unidentified, but anti-GBM positivity threshold set at >12 U/ml in the lab)*	30	Yes	Yes	After 6 sessions of hemodialysis	Yes (class IV LN)	Yes	LN and IAH	Anti-DNA positivity and hypocomplementemia (negative ANA)	Methylprednisolone (three boluses) and cyclophosphamide/ *stabilization of the renal function (at preterminal renal failure stage) and IAH decrease*
*K. Yamazaki et al*., 1993, *The Japanese Journal* (20)	Female *(Japan)*	58	*Unidentified*	*Unidentified*	Yes	Yes	*Unidentified*	Yes	No	Rash, photosensitivity, AIHA, LN	*Unidentified*	*Unidentified*

Unidentified: unidentified in the article; Article written in Japanese with only the abstract available in English.

ELISA, enzyme-linked immunosorbent assay; IFI, indirect immunofluorescence assay; ANA, antinuclear antibodies with titer >1/160, detected using immunofluorescence; IAH, intra alveolar hemorrhage; AIHA, autoimmune hemolytic anemia; AITP, autoimmune thrombocytopenia.

Some experimental data have suggested that LN and anti-GBM-induced glomerulonephritis could share common dysimmunitary mechanisms, leading for example to the overexpression of the HLA DRB15*01 antigen (21), a lack of regulation of T- lymphocyte populations (23), a pro-inflammatory cytokine environment (IL-17, IL-23) (24), or identical mutations in kallikrein genes (22). However, most of the studies published on anti-GBM antibodies are contradictory and yet support our results. Anti-GBM antibodies are known to be most often negative in the general Caucasian population, in the absence of vasculitis (10, 11). Our large study extends this idea to the SLE patient population by showing that these antibodies are negative regardless of the status “with or without LN” in SLE patients. The results of our study are in agreement with those of other studies demonstrating the high sensitivity and specificity of anti-GBM antibodies for the diagnosis of anti-GBM vasculitis. For example in Sinico RA and al, two SLE patient sera tested with four different anti-GBM antibody commercial kits were negative regardless of the technique used (29), and antibody specificity was between 90 and 100%. More recently, Bentov et al. tested 56 SLE sera for anti-GBM antibodies, but none of them returned positive (42). The specific pathogenicity of anti-GBM antibodies has also been extensively demonstrated (28, 43). Our study may have important implications for clinical practice as it suggests that physicians should not systematically order anti-GBM antibody testing for SLE patients, even in the presence of renal impairment. Conversely, and by extension with the current general population recommendations, anti-GBM antibody testing is critical in clinical presentations suggestive of anti-GBM vasculitis (intra-alveolar hemorrhage, rapidly progressive renal failure, etc.). Positivity should, even in case of known SLE and/or LN disease, lead the physician to consider vasculitis and thereby proceed with specific investigations (such as renal biopsy and/or thoracic explorations) to confirm the diagnosis and rapidly adapt therapeutic management. Our study underlines the central role of anti-GBM antibodies (as specific biomarkers) in the diagnostic strategy of vasculitis in the SLE and LN patient population.

Several hypotheses can explain the difference between our results and those of the Chinese study (9). First, the anti-GBM detection method used was not the same (EUROIMMUN^®^ in the Chinese cohort versus the EliA^®^ technique in our center), and disparities between the different kits used have already been shown to exist (29, 30). On the other hand, ethnic differences between the two SLE populations, who do not share the same biological and immunological characteristics, must be taken into consideration (44). In our cohort, the majority of sera came from Caucasians whereas all of the lupus patient sera came from Asians in the 2006 study. In fact, several studies have shown that proliferative glomerulonephritis was more frequent in Asian patients (45–47) than in Caucasians. The serum auto-antibody profiles also differ according to the patient’s ethnic origin. It should also be noted that in the Chinese study (9), only 42 patients in total had undergone renal biopsy, *versus* 100 in our study. A limitation of our study is that for SLE patients with LN, serum was not always collected at the time of lupus nephritis (LN). Consequently, we cannot exclude the possibility that some SLE patients may have developed anti-GBM antibodies at the time of LN, and that these antibodies could have disappeared following immunosuppressive therapy.

We believe our cohort is representative of the European SLE-patient population. On the clinical level, besides the renal damage, the main differences between the two patient groups (with or without LN) were the incidence of serositis and the average SLEDAI score. This correlation between serositis and LN has already been reported in other studies, such as the Le Thi Huong et al. cohort (5), or in a more recent study (2017) published on an Asian cohort of 1,526 SLE patients (48). LN patients’ SLEDAI score was higher than that of the SLE patients without LN and this result is in accordance with most cohorts, which report that renal damage most often occurs when the disease is still active (49, 50). On the immunological level, Alba et al. along with other teams, like us, have reported that anti-DNAs antibodies were more frequently positive in the case of renal damage [68 *versus* 50%, on their cohort of 127 LN patients (histologically proven) and 206 SLE patients without LN; p = 0.02; OR 2.35 (1.83; 4.03)] (51). In the end, the most surprising element in our cohort, was the low percentage of patients on hydroxychloroquine (only 64% of patients in the LN group, and 72% of those in the group without LN) despite current recommendations (52–54).

In this retrospective study, performed thanks to a Franco-German biobank of SLE patient sera, no significant correlation was found between SLE, LN, and serum anti-GBM antibody positivity. This study therefore supports the idea that anti-GBM antibodies are usually negative in a predominantly Caucasian lupus population, including in the presence of LN, and that systematic testing does not thereby seem useful in current practice, despite the report in the literature of some cases of borderline forms between SLE and anti-GBM vasculitis. Conversely, positive anti-GBM serum detection, even in patients with known SLE, should encourage physicians to consider a vasculitis diagnosis, and thereby lead to appropriate and timely investigation.

## Data Availability Statement

The raw data supporting the conclusions of this article will be made available by the authors, without undue reservation.

## Ethics Statement

The studies involving human participants were reviewed and approved by the French Ethical Research Committee—“Comité de Protection des Personnes” (CPP Est 2.02.2011). For the use of healthy volunteer blood donor sera, the study was approved by a regional ethics committee (CPP Sud-Est IV 16.03.2018). The patients/participants provided their written informed consent to participate in this study.

## Author Contributions

NB, principal investigator and author of the study. PO, investigator and co-author of the study. DG, laboratory assays. GG, laboratory assays. TT, laboratory assays. MT, laboratory assays. JC, statistical analyses. MD, statistical analyses. GS-B, syntax proofreading. BP, laboratory assays. TM, Goodlupus project coordinator with the Scientific committee of LBBR biobank. J-LP, investigator and co-editor of the study. AmS, investigator and co-editor of the study. Co-authors on behalf of the Scientific committee group of LBBR: TM, ZA, LA, H-ML, GB, BB, NM-B, SR, RV, OH, AnS. All authors contributed to the article and approved the submitted version.

## Conflict of Interest

The authors declare that the research was conducted in the absence of any commercial or financial relationships that could be construed as a potential conflict of interest.
